# Ensuring equity: Pharmacogenetic implementation in rural and tribal communities

**DOI:** 10.3389/fphar.2022.953142

**Published:** 2022-09-13

**Authors:** Tianna M. Leitch, Shayna R. Killam, Karen E. Brown, Kirk C. Katseanes, Kathleen M. George, Corbin Schwanke, Joshua Loveland, Abdallah F. Elias, Kerry Haney, Kate Krebsbach, LeeAnna I. Muzquiz, Susan B. Trinidad, Erica L. Woodahl

**Affiliations:** ^1^ L.S. Skaggs Institute for Health Innovation, University of Montana, Missoula, MT, United States; ^2^ Shodair Children’s Hospital, Helena, MT, United States; ^3^ Partnership Health Center, Missoula, MT, United States; ^4^ Tribal Health Department of the Confederated Salish and Kootenai Tribes, St. Ignatius, MT, United States; ^5^ Department of Bioethics and Humanities, University of Washington, Seattle, WA, United States

**Keywords:** pharmacogenetics, pharmacogenomics, implementation, rural, underserved populations, health equity, telehealth, telemedicine

## Abstract

Implementation strategies for pharmacogenetic testing have been largely limited to major academic medical centers and large health systems, threatening to exacerbate healthcare disparities for rural and tribal populations. There exists a need in Montana (United States)—a state where two-thirds of the population live in rural areas and with a large proportion of tribal residents—to develop novel strategies to make pharmacogenetic testing more broadly available. We established partnerships between University of Montana (UM) and three early adopter sites providing patient-centered care to historically neglected populations. We conducted 45 semi-structured interviews with key stakeholders at each site and solicited participant feedback on the utility of a centralized pharmacogenetic service at UM offering consultations to patients and providers statewide *via* telehealth. For settings serving rural patients—tribal and non-tribal—participants described healthcare facilities without adequate infrastructure, personnel, and funding to implement pharmacogenetic services. Participants serving tribal communities stressed the need for ethical practices for collecting biospecimens and returning genetic results to patients, largely due to historical and contemporary traumas experienced by tribal populations with regard to genetic research. Participants expressed that pharmacogenetic testing could benefit patients by achieving therapeutic benefit sooner, reducing the risk of side effects, and improving adherence outcomes for patients with limited access to follow-up services in remote areas. Others expressed concern that financial barriers to pharmacogenetic testing for patients of lower socioeconomic status would further exacerbate inequities in care. Participants valued the role of telehealth to deliver pharmacogenetic consults from a centralized service at UM, describing the ability to connect providers and patients to resources and expertise as imperative to driving successful pharmacogenetic implementation. Our results support strategies to improve access to pharmacogenetic testing for neglected patient populations and create opportunities to reduce existing healthcare inequities. By exploring critical challenges for pharmacogenetic implementation focused on serving underserved communities, this work can help guide equitable frameworks to serve as a model for other resource-limited settings looking to initiate pharmacogenetic testing.

## Introduction

Growing evidence supports the use of pharmacogenetic-guided medication management, yet adoption into standard practice has thus far been primarily limited to academic medical centers and large health systems serving urban patients (Fohner et al., 2019). Consistent with the diffusion of innovations theory—which postulates that extended periods of time are required for new health innovations to be widely disseminated—patients receiving care in rural primary care settings are often last to receive new treatments, care strategies, and the benefits of new health services (Dearing and Cox, 2018). Examples of this variable diffusion—or failure to integrate innovations into health systems serving neglected populations—have been observed for a variety of modern medical technologies (Boscoe and Zhang, 2017; Balas and Chapman, 2018; Hirko et al., 2020; Banbury et al., 2021; Chunara et al., 2021). Efforts to broadly democratize pharmacogenetic testing have faced barriers, with limited examples of pharmacogenetic testing implementation in rural and tribal healthcare settings (Dorfman et al., 2015; Dressler et al., 2019a; Dressler et al., 2019b; Stegelmeier et al., 2020).

Unique challenges impact access to health services in rural, underserved, and resource-limited communities. Examples include cultural or financial barriers to care, underdeveloped public transportation, and inadequate broadband internet access that hinder the implementation of telehealth strategies (Douthit et al., 2015; Hirko et al., 2020). Additionally, despite ongoing federal and state efforts to incentivize professionals to serve rural areas, such communities face an ongoing shortage of physicians and trained health professionals (Health Resources and Services Administration 2022a). While medically underserved designations have helped to establish and maintain health services for groups frequently facing barriers to healthcare, disparities have continued to permeate health systems and access to specialty services remains scarce (Centers for Disease Control and Prevention 2013).

In addition to barriers faced by patients living in poverty or in geographically isolated locations, some Americans continue to experience significant health disparities in relation to race and ethnicity. As highlighted by the COVID-19 pandemic, specific racial and ethnic groups experience disproportionately high rates of severe COVID-19 illness, particularly African American, Hispanic Americans, and American Indian and Alaska Native (AIAN) people, further emphasizing longstanding health disparities (Carethers, 2021). This trend is true for many conditions, with AIAN people continuing to face disproportionate disease burden for several chronic illnesses including diabetes, liver disease, and respiratory diseases, and have lower overall life expectancy rates than the general population (Centers for Disease Control and Prevention 2013; Indian Health Service 2019).

Recent studies have demonstrated that healthcare providers anticipate an increase in the use of pharmacogenetics-guided prescribing in the near future (Olander et al., 2018; Hundertmark et al., 2020; Rahawi et al., 2020; Vest et al., 2020). Even so, many still consider pharmacogenetic testing a “luxury” service rather than a critical clinical decision-making tool. In urban settings, fiscal barriers tend to delay implementation and these concerns are only amplified in rural settings (Verbelen et al., 2017; Hockings et al., 2020). Additionally, a well-documented shortage of genetic specialists in the United States demonstrates ongoing demand for professionals with training to deliver and interpret genetics-related services (Maiese et al., 2019; Chou et al., 2021; Schaaf, 2021). Given the challenges that many underserved communities face, the potential of pharmacogenetic testing to optimize medication therapy in a timely manner is compelling. Their exclusion from pharmacogenetic research and implementation efforts may exacerbate healthcare disparities. Additionally, pharmacogenetic research has largely included participants primarily of European ancestry, with knowledge of pharmacogenetic variation in diverse populations desperately lagging (Popejoy and Fullerton, 2016; Martin et al., 2019; Fatumo et al., 2022).

To overcome the unique barriers to implementation in rural and tribal settings, it is imperative that creative solutions are developed to ensure pharmacogenetic testing is integrated into practice for all patients. Our goal is to inform the development of a pharmacist-led pharmacogenetic implementation strategy based at University of Montana (UM) focused on serving neglected patient populations, and subsequently, to inform implementation in rural areas. Montana is populated by diverse, underserved patient groups, including rural and tribal populations, patients of lower socioeconomic status, and patients with limited access to care in sparsely populated rural regions (Health Resources and Services Administration 2019; Montana Department of Health and Human Services 2019; Montana Department of Commerce 2020; United States Census Bureau 2020). Montana—the fourth largest state in the United States—at approximately 147,000 square miles, boasts a population density of just 6.8 persons per square mile with two of every three residents living in rural areas (Montana Department of Health and Human Services 2019). In total, 55 of 56 counties in Montana have received some form of HPSA designation (Health Resources and Services Administration 2022b). Montana is also home to 12 Tribal nations and AIAN peoples are the largest minority group in the state making up almost 7% of the population (Montana Office of Public Instruction 2020; United States Census Bureau 2020).

We have engaged three partner sites in Montana focused on providing patient-centered care to historically neglected populations. The Tribal Health Department of the Confederated Salish and Kootenai Tribes (CSKT)—with whom we have a long-standing community-academic research partnership in pharmacogenetics (Boyer et al., 2011; Woodahl et al., 2014; Morales et al., 2016)—provides a network of primary care health and wellness services to patients with Indigenous ancestry throughout the Flathead Reservation in Montana (Tribal Health Department of the Confederated Salish and Kootenai Tribes 2022). Partnership Health Center (PHC)—a federally qualified health center in Missoula, Montana—provides a variety of healthcare services and seeks to provide equitable, comprehensive care for insured or uninsured patients of all income levels (Partnership Health Center 2022). Shodair Children’s Hospital (Shodair) in Helena, Montana serves children and adolescents throughout the state providing acute, residential, and outpatient psychiatric care (Shodair Children’s Hospital 2022). With our partners, we seek to integrate pharmacogenetic testing services across a broad range of therapeutic applications utilizing telehealth to overcome obstacles of geographic remoteness.

Our study aims to identify unique facilitators and barriers to pharmacogenetic implementation among disadvantaged, underrepresented, and neglected populations. The data collected through semi-structured interviews will inform a needs assessment for future implementation efforts, specifically tailored to improve access to pharmacogenetics among patient populations experiencing significant health disparities. While participants across all three practice sites shared perceptions regarding the utility of pharmacogenetic testing for a variety of disease states and medication management concerns, most providers shared previous experiences and exposures to pharmacogenetic testing within the context of psychiatric care. Our goal is to also evaluate stakeholders’ perceptions of using an innovative telehealth model to implement a pharmacist-led pharmacogenetic program statewide. We expect that the provision of a centralized resource operating *via* a “hub and spoke” model may expand access to pharmacogenetics for rural, underserved, and tribal patients, creating equitable frameworks for delivery in other resource-limited health systems.

## Materials and methods

### Study setting

We completed interviews with healthcare professionals, administrative staff, and informatics professionals at three different sites (CSKT, PHC, and Shodair) serving underserved and neglected populations in Montana (Table 1). Each partner site offers a mixture of primary care and specialized treatment and seeks to provide ethical and equitable care for patient populations experiencing health disparities. This work was approved by UM and Salish Kootenai College Institutional Review Boards.

**TABLE 1:** Partner site descriptions.

	Confederated Salish and Kootenai Tribes (CSKT)	Partnership Health Center (PHC)	Shodair Children’s Hospital (Shodair)
General Descriptor:	A network of healthcare clinics serving the CSKT based out of St. Ignatius, Montana.	A federally-funded community health center located in Missoula, Montana.	A pediatric psychiatric hospital located in Helena, Montana, also providing the state’s only comprehensive medical genetics services.
Services Offered:	• Medical	• Medical	• Acute and residential psychiatric services
	• Dental	• Dental	• Outpatient psychiatric care
	• Pharmacy	• Pharmacy	• Medical genetics services
	• Behavioral health	• Behavioral health	• CLIA certified diagnostic genetics laboratory
	• Telehealth services	• Telehealth services	• Telehealth services
	• Physical therapy		• Newborn screening follow-up
	• Optical		
	• Audiology and Speech		
	• Community Health		
Primary Patient Populations:	Tribal Health Clinics focus on providing high-quality care to recipients “grounded in Tribal values” for the CSKT	PHC serves 15,000 patients in Missoula County	Psychiatric services for children and adolescents throughout Montana
	• 11,000 eligible recipients that include CSKT members and descendants	• Approximately 52% of patients face economic insecurity	• Approximately ∼4,000 patients served in 2020
	• Provides care to members of other federally-recognized tribes who reside on the Flathead Reservatio	• 29% of patients live at or below the federal poverty level	• 70% children from families living at or near poverty
		• 7% of patients served identify as homeless	• 39% of admissions referred from rural areas
		• 17% of patients are uninsured	• 14.7% of admissions identified as American Indian
			• Medical genetics program serves prenatal, pediatric, and adult populations

### Study design, data collection, and analysis

We conducted interviews to elicit stakeholders’ perceptions, attitudes, and opinions regarding pharmacogenetic implementation in rural settings as well as potential impacts on patients and populations served. Partners at each site (CS, JL, AFE, KH, KK, and LIM) helped identify prospective participants, which generated referral sampling within each location. Eligibility criteria were that participants be ≥18 years old and working at the partner site. Participants were not presented any background information about pharmacogenetics prior to the interview. Interviews at all sites were halted when theoretical saturation was achieved (Starks and Trinidad, 2007).

For interviews conducted at PHC and Shodair, we developed an original semi-structured interview guide utilizing components of the Consolidated Framework for Implementation Research (CFIR) (Damschroder et al., 2009). The guide was developed using the CFIR Interview Guide Tool and included 11 open-ended questions spanning three categories: medication therapy management, site priorities and outcome measures for pharmacogenetics, and a needs assessment to begin a pharmacogenetics testing program. These categories were modeled on CFIR evaluation domains of intervention characteristics, outer setting, inner setting, characteristics of individuals, and process. Participants were also asked to complete a brief pre-interview survey to collect demographic information and general experience with pharmacogenetics *via* Qualtrics (Provo, UT, Unites States). Interviews lasted 30–60 min and were conducted in person or *via* telephone by members of the research group (TML, SRK, KCK, and ELW) from August to October 2019.

For interviews conducted at the CSKT Tribal Health Department, we conducted a secondary analysis of data from a prior formative study concerning provider perceptions of precision medicine, of which pharmacogenetics is a component. Interviews were semi-structured, lasted 30–60 min, and were conducted over the telephone by a member of the research group (SBT) in April 2019. The CSKT interviews did not collect participant views regarding telehealth for pharmacogenetic consultation, therefore, those results are not reported.

Interviews were audio-recorded and transcribed for a descriptive thematic analysis to identify major themes generated from participants’ knowledge, attitudes, and beliefs (Sandelowski, 2000). Interview transcripts were uploaded into ATLAS.ti (Berlin, Germany). TML and SRK read transcripts and worked together to iteratively develop a codebook. The resulting codes and themes were independently evaluated by the full research team and were subsequently analyzed for any remaining discrepancies or sources of potential bias. TML, SRK, and KMG then finalized the codebook and applied it to the full dataset, meeting to resolve differences as needed.

## Results

### Overview

Across the three sites, 45 participants were recruited and interviewed (Table 2). Interviews included participants with a variety of clinical, administrative, and informatics expertise as implementing new interventions affects not only clinical workflow, but also that of other staff. Major themes are highlighted below with sections beginning with a summary of findings that were common across sites followed by findings unique to each. Direct quotations provide evidence for each major theme (Tables 3–6).

**TABLE 2:** Participant demographics (*n* = 45).


Characteristics	Mean (range)
Age (years)	44 (28–73)
Years in Practice^a^	12 (1.5–44)
Gender	*n* (%)
Female	30 (67.7%)
Male	15 (33.3%)
Stakeholder Role	*n* (%)
Physician	14 (31.1%)
Nurse Practitioner	8 (17.8%)
Pharmacist	6 (13.3%)
Physician Assistant	1 (2.2%)
Administration	5 (11.1%)
Information Technology	2 (4.4%)
Informatics	1 (2.2%)
Other^b^	8 (17.8%)
Experience with Pharmacogenetics^c^	*n* (%)
Agreed with the statement “I am familiar with pharmacogenetic testing”	20 (64.5%)
Previously ordered a pharmacogenetic test	10 (32.3%)

a Applicable only to clinical participants.

b Includes roles of molecular technologist, genetic counselor, dietician, registered nurse, lab technician, and medical records.

c Only participants from Shodair and PHC were queried about their experience with pharmacogenetics (*n* = 31).

**TABLE 3:** Opportunities to mitigate medication management concerns unique to neglected populations.

Confederated Salish and Kootenai Tribes (CSKT)	Partnership Health Center (PHC)	Shodair Children’s Hospital (Shodair)
“The dissemination of information from research centers to the frontlines of care delivery, especially in a frontier state like ours, is always a challenge, whether it’s precision medicine or just [the] latest cancer protocols.”—CSKT01, Physician	“I would say that our mission and vision is to provide high quality healthcare to everyone … the majority of our patients are folks who have a lot of struggles that they deal with on a daily basis, mostly socioeconomic-related, so whether they don’t have insurance, they’re homeless, the insurance they do have is really limited, they can’t afford a lot of the premiums for certain treatment, and so breaking down those barriers is a huge part of the vision here.”—PHC01, Pharmacist	“Sometimes when kids get here, they’re on a lot of different medications, and so trying to get that down to a reasonable amount, whatever that might be [is the challenge]. You can imagine [the difficulty of] a seven-year-old trying to take seven different medications. Maybe with the right management, it could be [reduced to] three or four.”—Shodair01, Administrator/Physician
“I think that there’s … a misrepresentation [that current guidelines can be universally applied to people of all ancestries] in … both the [pharmacogenetics] research and also the literature of both management options and then also barriers that some [historically disadvantaged] communities face.”—CSKT02, Physician		“A hard part that we see is kids leave here and they go back to rural communities especially, but even larger communities … some of the newer medications for some [patients] are kind of—I might use the word “scary” for some of our rural providers especially, and so they are nervous about prescribing those, so we try to give them guidance on [those medications].”—Shodair03, Administrator

**TABLE 4:** Potential barriers to pharmacogenetic implementation for underrepresented populations.

Confederated Salish and Kootenai Tribes (CSKT)	Partnership Health Center (PHC)	Shodair Children’s Hospital (Shodair)
“Some people feel that [genetics] is a potentially sensitive area, that maybe either people who shouldn’t have access to that information might get access to that information or that people who are ‘qualified researchers’ may nonetheless ask research questions that are offensive to certain communities.”—CSKT04, Pharmacist	“To our patient population, cost is always an important thing, so we have a tendency not to run tests unless they’re going to be meaningful and make a difference in care. We don’t want to be doing tests that are unnecessary or tests that have so many limitations that they’re not useful.”—PHC02, Physician/Administrator	“That’s probably one reason why I haven’t used [pharmacogenetics] is because I don’t feel like I could—as a family nurse practitioner, I didn’t get specific training on it, so if I didn’t precept or have somebody to work with who understood it well, there’s no reason for me to order it.”—Shodair06, Nurse Practitioner
“I think working primarily with a native population and recognizing some of the research that has already been done, and I know first-hand that some of those genetic indicators are more represented in [the] Caucasian population so then the benefit of that technology has been to that larger [European] population.”—CSKT02, Physician	“For a lot of people whose clinical life is already so complicated and hard and time-consuming, it’s really stressful to think about adding something new, so I think just being really thoughtful about integrating it in a way that is going to seem palatable to that spectrum. […] Sometimes, it’s just like, ‘I can’t deal with this other new thing’.”—PHCO3, Physician	“One thing I have heard our physicians complain about is the time it takes for results … especially on the acute units. If the kids are there for 7 days, if it takes a week to get [test result] back … they’re gone. It’s pointless.”—Shodair01, Informatics
		“One of the things is I would want to make sure that [pharmacogenetics is] available for all patients. Whenever we’re making decisions, treatment decisions based on insurance, that doesn’t feel good.”—Shodair03, Physician

**TABLE 5:** Facilitators and perceived value of pharmacogenetic testing services targeted to underserved patient populations.

Confederated Salish and Kootenai Tribes (CSKT)	Partnership Health Center (PHC)	Shodair Children’s Hospital (Shodair)
“I do think [pharmacogenetic testing] has a place, particularly … for management of depression. My thoughts going forward is that I think it’s a great opportunity. It’s like all technology gets cheaper the longer we use it. If we can really dial in what it takes to get a chronic disease under control, whether it’s diabetes or treat their colon cancer, I think it will be well received… I also think if you can really tailor medical therapy to be effective and, of course, we’re going to improve health outcomes, which makes a lower cost of care, and less unexpected interactions within the medical system.”—CSKT02, Physician	“Providing information about which test to order, to me, is [very] valuable because there’s a lot of tests out there. And it’s unclear to me which are most evidence-based, which are validated, and which provide clinically valuable information. If I’m going to order a [pharmacogenetic] test, I want to know what to do with those results. And I want that knowledge to enable me to make a decision that I wouldn’t have been able to make otherwise or wouldn’t have felt as good about making without that information.”—PHC05, Administrator/Physician	“Ideally, you see maybe a faster time to effective dose or maybe less trials before you get to a treatment that works really well. Those would be good outcomes. Maybe you could even look at hospital days [length of stay] That would be cool. I think those are the kinds of things that then you’re talking people’s language because you’re saving money.”—Shodair04, Genetic Counselor
“I’m all for minimizing medicine [polypharmacy] as best we can… That’s kind of my end goal as a pharmacist.”—CSKT05, Pharmacist	“The providers have to buy into it, that this would help make their practice better, enhance their practice, and see the benefit of how it would -- and then the rest of us could get onboard.”—PHC04, Administrator	“I think rather than an entire [pharmacogenetics] report—because based on the ones I’ve seen previously and I’m sure this [new service] is a different test—that could end up being quite a stack of paper with all the recommendations. So I would like to see the recommendations first [and then details]. Of course, it would be nice if [pharmacogenetic test results] could just be uploaded into [the EHR]. I think that would make it easy for everyone to access.”—Shodair09, Physician

**TABLE 6:** Role of a unique telehealth delivery model for pharmacogenetic consultations.

Confederated Salish and Kootenai Tribes (CSKT)	Partnership Health Center (PHC)	Shodair Children’s Hospital (Shodair)
Not addressed (secondary analysis)	“I really think that this type of a concept [telehealth consultation service] with the center in our state makes sense, and I think it makes sense to house it at the University of Montana. I think it’s a really important opportunity that we need to be exploring … in five or 10 years it’s going to be very important… I think it’s good for us to be ahead of the curve and start exploring this now.”—PHC06, Pharmacist	“I think looking at providers around Montana, a [desirable] outcome for me would be the opportunity to become a really valuable resource to rural frontier providers so that they can refer to us [partnership between Shodair and University of Montana] for [pharmaco]genetics consults. But, if they can refer for pharmacogenetic testing [at Shodair], and if we can do it in a timely, cost-effective manner so they can make treatment decisions, then we become an invaluable partner to them.”—Shodair05, Administrator
	“Offering sometimes a hub for information is really helpful … that kind of warm line that we can call and say like, “Oh, this is the situation. This is my question.” I think having that kind of point of care resource is really helpful.”—PHC05, Administrator/Physician	

### Opportunities to mitigate medication management concerns unique to neglected populations

Participants across sites shared concerns regarding polypharmacy and the potential financial burden of medication therapies for their patients (Table 3). They cited goals of simplifying medication management for their patients on more complex regimens and limiting potential drug interactions. Additionally, participants described the importance of maximizing medication therapies in a time-sensitive manner as a primary treatment goal, highlighting their concern for the lack of access to adequate follow-up care that their patients often face. For these underserved populations, a breadth of health inequities presents difficulties in medication management and the vast distances between sparsely populated communities in Montana further complicates appropriate follow-up.

In addition to challenges in medication management for patients in rural communities, participants who serve tribal patients noted specific barriers. As a health system focused on providing care grounded in tribal values for any and all members of federally recognized tribes, CSKT Tribal Health providers were concerned that the clinical implementation of new medication therapies or treatment strategies in rural practice settings is often delayed, which negatively impacts tribal communities. Several participants described this phenomenon as a challenge in their everyday practice. CSKT participants noted that when new innovations are made available, they are seldom trialed adequately in all populations. For example, some providers expressed concerns that treatment options for many chronic conditions lack evidence to support their use in non-European descended populations. Participants opined that many aspects of modern medicine and its practice fail to adequately address a variety of clinically important factors, including a host of environmental or genetic factors, as well as historical and present barriers to accessing care for tribal patients.

Participants at PHC serve patients who experience inequitable access to healthcare and a variety of barriers to medication management. PHC participants heavily consider barriers their patients face—including poverty, homelessness, and being members of minoritized or marginalized groups—when developing treatment plans. Medication therapies and strategies are adjusted to help address specific concerns such as cost, monitoring, and access. As the patients treated at PHC may have a variety of the aforementioned financial, environmental, or social factors influencing their care and treatment plans, optimizing medication therapies and reducing barriers to access or improving medication therapy outcomes were primary concerns for healthcare professionals.

Participants at Shodair primarily serve another one of Montana’s most vulnerable populations, children and adolescents undergoing psychiatric treatment, as well as serving as the primary hub for medical genetics resources and services throughout the state. Participants spoke about the difficulty of managing pediatric patients on a variety of psychiatric medications, who often undergo multiple medication changes—a process that can take months to years—until they reach therapeutic stability. Many patients at Shodair are initially admitted to the acute inpatient unit and subsequently transitioned to outpatient care following adequate symptom management. Providers described concerns regarding the timeliness of achieving therapeutic drug plasma levels and subsequent desired response; many psychiatric medications require trial periods of 2–4 weeks before symptom improvement, yet the typical acute inpatient stay is 7–10 days, making initial treatment selection especially critical. Additionally, providers cited challenges in successfully transitioning patients from the hospital setting back to community-based care located elsewhere in Montana, considering many patients return to rural or tribal communities with limited access to specialty psychiatric care. Providers stated that due to the shortage of psychiatric resources and care options for pediatric patients in geographically isolated communities, changes to medications or treatment strategies initiated at Shodair are not always effectively continued or monitored following discharge.

### Potential barriers to pharmacogenetic implementation for underrepresented populations

Across all sites, interviewees touched on the expected concerns surrounding the cost of pharmacogenetic testing services, reimbursement for the tests and consultations of results, education and buy-in for providers unfamiliar with pharmacogenetics, and anticipated challenges with the integration of results into the electronic health record (EHR) to assist with clinical decision support (Table 4). Based on the unique make-up of the patient populations served, specific barriers to successful implementation became central within interviews amongst each respective site.

Providers at CSKT Tribal Health reported their patients may have mistrust in genetics, including pharmacogenetic testing and research, given historical misuse and abuse of genetic data from AIAN peoples. In the context of experiences with data stewardship in genetics research, participants noted that the majority of research completed to date has failed to adequately address concerns within AIAN populations regarding discrimination, stigma, and other potential harms. Some providers considered that existing evidence may not be applicable to AIAN populations due to the lack of ancestral diversity in research used to generate pharmacogenetic testing arrays and testing guidelines.

Interviewees at PHC identified cost of healthcare services as the primary barrier to their patients. Many participants described concerns regarding the prioritization of testing among those patients already facing a variety of sociodemographic factors that impact access to care and how to determine which patients could benefit most from the added expense of pharmacogenetic testing. Concerns around healthcare equity were a strong theme at PHC and providers speculated on how to ensure that testing was an option for all patients, not only those who could afford it. Several participants anticipated that there may be hesitancy from practitioners who believe the return on investment from pharmacogenetic testing remains inadequate, particularly within a resource-limited practice setting. Sociodemographic challenges already limit access to basic preventative health or primary care services for many PHC patients. Resource constraints lead to hesitancy toward health innovations that are not yet accepted as standard of care among PHC providers. The risk of overburdening providers with more information or further complicating workflow was apparent among participants.

Shodair participants identified the turnaround time for testing results to be made available to practitioners as a critical concern. With return of results often taking at least a week, many felt that pharmacogenetic testing would be of limited value in the acute, inpatient setting where patients are typically discharged within 7–10 days. Participants described that testing may therefore have greater utility in the outpatient setting, with providers following patients over a longer period of time. Participants also identified the successful integration of pharmacogenetic testing results in the EHR as a key factor in achieving provider buy-in and perceived utility. Informatics participants noted that Shodair currently lacks a location or protocol for the standardized storage of pharmacogenetic testing results and emphasized the need to ensure security in inter-facility data transfers, particularly for genetic information. There were concerns that even if the testing were utilized, many providers—especially those without specialized training in pharmacogenetics—would not feel comfortable interpreting results themselves and using them to guide prescribing decisions.

### Facilitators and perceived value of pharmacogenetic testing services targeted to underserved patient populations

Many individuals interviewed shared positive perceptions of the ability of pharmacogenetic testing services to help achieve therapeutic benefit and reduce time to effective dose (Table 5). Participants across all sites identified additional training, education, and resources for staff as a significant facilitator to implementation. Perceived benefits that were noted across all sites included reduced risk for adverse drug events related to patient phenotype status and improved medication management outcomes for patients with limited access to follow-up services due to social, environmental, or financial barriers.

A primary facilitator of an implementation effort highlighted by CSKT providers was the long-standing partnership and engagement fostered between the CSKT and UM researchers, built on more than a decade of ongoing pharmacogenetic research. Healthcare stakeholders interviewed at CSKT shared positive perceptions regarding the clinical utility of pharmacogenetic testing. Practitioners and health professionals generally agreed pharmacogenetic testing could support individualized, targeted treatment for their patients and could be utilized to minimize or reduce preventable adverse reactions related to medications. Several participants described potential benefits in helping to minimize risks of polypharmacy as a result of more targeted dosing strategies.

Participants at PHC prioritized achieving provider buy-in and ensuring that providers have sufficient education, training, and point-of-care resources. Participants suggested that identifying pharmacogenetics “champions” within the organization would serve as a key facilitator of successful implementation. Several participants pointed to established PHC protocols for initiating new clinical services and emphasized the importance of engagement with all departments. New programs at PHC are generally piloted in a smaller area of the clinic, where major barriers and concerns can be addressed quickly and without impact on the entire clinic workflow. Programs that perform well in the pilot are then expanded to other departments. PHC participants prioritized educational opportunities for staff members as key to successful implementation. Several participants recommended that education—including presentations from experts in the field and connecting with practitioners as “point-of-contact” resources—would facilitate pharmacogenetic testing.

Shodair participants, particularly those in informatics, emphasized the importance of effective integration of pharmacogenetic testing results into the EHR and clear channels of communication between Shodair and the UM pharmacogenetic consultation service exhibiting secure and protected data sharing. Providers at Shodair prioritized an approach that would expedite the timeline to effective medication management and more targeted therapy, which they believed could generate better post-discharge outcomes for patients, including reduced rates of readmission. Participants felt that an implementation effort in an outpatient setting would provide better opportunities for reimbursement and more flexibility in turnaround time of testing results.

### Role of a unique telehealth delivery model for pharmacogenetic consultations

During interviews, a telehealth delivery model offered by pharmacists and pharmacogenetic experts based at UM was introduced as a means to achieve equitable pharmacogenetic testing implementation (Table 6). As described above, participants at PHC and Shodair were questioned on their opinions of utilizing telehealth technology to provide pharmacogenetic consultations on test results and education to providers on the use of pharmacogenetics. These questions were not addressed within interviews completed with CSKT participants as the results from the CSKT were from a secondary analysis of data from a previous study that did not address telehealth. Participants expressed positive perceptions of using telehealth for the return of pharmacogenetic results and valued a service that could connect providers to resources and expertise without requiring significant changes to provider education and workflow on-site. Participants preferred that testing results be integrated into the EHR and providers receive both result interpretations and treatment recommendations. Providers also discussed the importance of having pharmacogenetic experts available for future clinical support and education, as pharmacogenetics-driven prescribing guidance continues to evolve. Participants spoke of the potential benefits of increasing access to specialized pharmacogenetic testing for populations who would otherwise have to travel long distances to access this expertise.

Participants at PHC identified the importance of having a centralized resource for pharmacogenetic recommendations, guidance, and support as a major benefit for this site, as well as other health systems providing primary care or behavioral health services across the state. Tasked with providing a great variety of primary care services, providers reported that integration of a new service like pharmacogenetic testing—with applicability across a range of specialties—would not be feasible for general primary care providers to manage independently without a tailored support system. The integration of a consultation service provided *via* a centralized resource was particularly attractive to PHC providers and several interviewees highlighted the importance of improving access to pharmacogenetic resources for individuals of all backgrounds. Participants shared positive perceptions regarding telehealth modalities for limiting costs while increasing access to these services for a variety of patient populations.

Participants at Shodair felt a telehealth model would serve as an appropriate strategy for successful integration of pharmacogenetic testing into outpatient services. Participants noted that additional, dedicated resources and personnel would be required to provide a pharmacogenetic testing service to Shodair providers and their patients. Therefore, interviewees identified a telehealth model as a potential alternative to provide patient-specific recommendations, guidance, resources, and ongoing education to providers. Participants felt that providing a pharmacogenetic service—not only to the providers located at Shodair, but also as a resource for providers across the state—could help to ensure the testing results are utilized well after patients are discharged.

## Discussion

Innovative clinical services—like pharmacogenetics—are often considered out of reach for patients in rural and tribal areas due to concerns regarding the sustainability and financial feasibility of new programs, perpetuating a troubling trend in which novel healthcare advances remain largely inaccessible for patient populations already experiencing significant health disparities. By failing to seek out unique solutions for pharmacogenetic implementation strategies in rural and tribal settings, existing health disparities may be exacerbated. For patient populations with limited access to care, high-quality medication management, access to pharmacogenetic testing, and appropriate follow-up are significant concerns among providers. Through qualitative interviews conducted with three early-adopter sites throughout the state of Montana, we found an interest in the use of pharmacogenetic testing to help address these concerns. These interviews demonstrated that facilities serving rural and tribal patients are uniquely situated to benefit from pharmacogenetic testing and pharmacist-led consultations delivered remotely *via* telehealth. Given well-established obstacles for geographically isolated and underserved communities, telehealth offers the ability to provide specialized and innovative clinical services to patients who may stand to benefit from targeted treatment strategies most.

At the CSKT Tribal Health Department—a health system providing care for those individuals of tribal ancestry—participants shared concerns regarding patient engagement and acceptance of pharmacogenetic testing given historical misuse and abuse of genetic data among Indigenous peoples. Within this context, providers and administrators at this site emphasized the importance of educational resources regarding pharmacogenetic testing for both healthcare providers and patients, providing special consideration and sensitivity for AIAN patient populations. Participants shared concerns that existing guidelines and research within the pharmacogenetics field have failed to adequately include Indigenous peoples and expressed a desire to continue fostering relationships of mutual trust between the CSKT Tribal Health, the patients they serve, and ongoing pharmacogenetic initiatives with researchers at UM.

At Partnership Health Center—a community health center that serves patients across the sociodemographic spectrum—participants’ concerns centered on the importance of developing a strategy for equitable implementation of pharmacogenetic testing. Participants identified equitable opportunity, in terms of both cost and physical access to education and counseling resources, as a primary barrier to widespread adoption. Additionally, as a site that offers comprehensive primary care services addressing a multitude of disease states, PHC participants highlighted concerns surrounding the shortage of specialized expertise regarding pharmacogenetic testing and lack of access to consultation services for primary care providers and patients in rural areas. Participants were enthusiastic for readily available resources and support through a UM-based centralized pharmacogenetic service and described it as a critical component of a successful implementation strategy.

As a site that serves as a leader and statewide resource for pediatric psychiatric services, providers at Shodair Children’s Hospital were familiar with both barriers and key facilitators of implementing pharmacogenetic testing services within their practice setting. Interviewees identified outpatient settings—in comparison to inpatient or acute care—as the ideal point for pharmacogenetic testing implementation and integration within existing workflows. Participants prioritized potential benefits of partnering with UM to provide pharmacogenetic consultations to advance the delivery of cutting-edge medication management initiatives to patients throughout the state. Participants at Shodair also valued a successful integration of pharmacogenetic test results into the EHR as an imperative step for therapy modifications and follow-up, particularly for patients transitioning from Shodair to their local community care settings.

In addition to these novel findings in rural, underserved, and tribal healthcare settings, participants also expressed themes that have been described in previous analyses of health professionals’ perceptions of pharmacogenetic testing and implementation feasibility (Dorfman et al., 2015; Klein et al., 2017; Olander et al., 2018; Rahawi et al., 2020; Stegelmeier et al., 2020; Vest et al., 2020). Common themes among all three sites included concerns regarding adequate turnaround time for testing results, concerns around cost and reimbursement of testing, and goals for successful integration of testing results into an EHR. Participants were generally positive regarding a pharmacogenetic implementation effort and considered decreased time to effective dose, minimizing the trial-and-error process of prescribing, and reducing adverse drug events as potential benefits. Participants at all sites valued pharmacogenetic testing results as a clinical decision support tool, but expressed concerns that the utility of the results may not be fully realized without significant education or clear guidance from experts in the field. Overall, participants involved in the analyses shared positive perceptions of a pharmacist-led consultation service at UM providing clinical recommendations and serving as a local resource for pharmacogenetic expertise.

Participants were enthusiastic that leveraging telehealth modalities could aid in pharmacogenetic implementation across a geographically expansive region and provide critical access to expertise otherwise unavailable to patients in rural and underserved areas. Telehealth has been proposed as a strategy to increase access to specialized services for rural communities and may be a valuable tool for pharmacogenetic implementation (Danylchuk et al., 2021; Westby et al., 2021). The COVID-19 pandemic has highlighted opportunities for telehealth to address healthcare disparities, however, the adoption is still lower in nonmetropolitan areas (Chen et al., 2021; Frydman et al., 2022). Access to high-speed internet is also a challenge for rural communities to take full advantage of the benefits offered by telehealth, although Montana is forward-thinking in improving and expanding high-speed internet for its citizens. In December 2020, the Federal Communications Commission awarded $125 million to Montanan firms to develop broadband infrastructure in rural regions as well as a providing internet licenses to the seven Tribal Reservations through the Rural Tribal Priority Window Initiative (Pfohl, 2020; Veigle, 2020). Additionally in February 2021, the Montana State Legislature unanimously voted to expand telehealth coverage requirements and remove site restrictions for services in response to rising trends in telehealth due to the COVID-19 pandemic (Montana State Legislature, 2021). These expansions demonstrate significant interest in utilizing telehealth to improve access to health services for Montanans and bodes well for our strategy to develop integrated pharmacogenetic telehealth solutions for stakeholders in rural, underserved, and tribal areas.

Limitations of our study include a small sample size and the possibility that themes may have been influenced by limited exposure to pharmacogenetic testing in health systems serving rural and underserved communities. Additionally, participants who consented to interviews may have been more likely to share positive perceptions about pharmacogenetics, therefore, negative opinions may be underrepresented as a result. While these limitations may influence generalizability to practices in metropolitan settings, we assert that our findings provide unique insights regarding perceptions of pharmacogenetic implementation efforts in resource-limited areas and may aid in the diffusion of pharmacogenetics to the benefit of neglected populations.

Our findings will inform an implementation strategy focused on improving access to pharmacogenetic testing for underserved and neglected patient populations, and increasing inclusion of underrepresented groups in pharmacogenetic research. Given well-established challenges to improving access to even baseline preventative health services in some rural, underserved, and tribal communities (e.g., shortage of health professionals, geographic remoteness, and economic insecurities), achieving equal access to care—especially for costly specialty services like pharmacogenetic testing—remains challenging. Through the development of a centralized “hub and spoke” pharmacist-led pharmacogenetic consultation service at UM, we will continue to explore critical challenges and facilitators for implementation strategies focused on serving underserved communities and the providers caring for them. By leveraging telehealth modalities for the dissemination and implementation of pharmacogenetics to underserved areas, our work may generate solutions that have wider utility and applicability for improving access to pharmacogenetic testing for patients living in a variety of resource-limited settings. We will pursue a pharmacogenetic implementation effort in an ethical and equitable manner through the development of a model focused on improving diffusion of pharmacogenetics to communities that have historically been the last to benefit from cutting-edge health innovations.

## Data Availability

The datasets presented in this article are not readily available because participant data from the Confederated Salish and Kootenai Tribes are under the stewardship of tribal sovereignty and available only with explicit Tribal approval. Requests to access the datasets should be directed to erica.woodahl@umontana.edu.

## References

[B1] Balas E. A. Chapman W. W. (2018). Road map for diffusion of innovation in health care. Health Aff. 37 (2), 198–204. 10.1377/hlthaff.2017.1155 29401030

[B2] Banbury A. Smith A. C. Mehrotra A. Page M. Caffery L. J. (2021). A comparison study between metropolitan and rural hospital-based telehealth activity to inform adoption and expansion. J. Telemed. Telecare 26, 1357633X2199820. 1357633x21998201. 10.1177/1357633x21998201 33765879

[B3] Boscoe F. P. Zhang X. (2017). Visualizing the diffusion of digital mammography in New York state. Cancer Epidemiol. Biomarkers Prev. 26 (4), 490–494. 10.1158/1055-9965.EPI-16-0928 28154106

[B4] Boyer B. B. Dillard D. Woodahl E. L. Whitener R. Thummel K. E. Burke W. (2011). Ethical issues in developing pharmacogenetic research partnerships with American Indigenous communities. Clin. Pharmacol. Ther. 89 (3), 343–345. 10.1038/clpt.2010.303 21326261 PMC3090734

[B5] Carethers J. M. (2021). Insights into disparities observed with COVID-19. J. Intern. Med. 289 (4), 463–473. 10.1111/joim.13199 33164230 PMC9325576

[B6] Centers for Disease Control and Prevention (2013). CDC health disparities and inequalities report - United States, 2013. Available: https://www.cdc.gov/mmwr/pdf/other/su6203.pdf (Accessed March 30, 2022).

[B7] Chen J. Amaize A. Barath D. (2021). Evaluating telehealth adoption and related barriers among hospitals located in rural and urban areas. J. Rural. Health 37 (4), 801–811. 10.1111/jrh.12534 33180363 PMC8202816

[B8] Chou A. F. Duncan A. R. Hallford G. Kelley D. M. Dean L. W. (2021). Barriers and strategies to integrate medical genetics and primary care in underserved populations: A scoping review. J. Community Genet. 12 (3), 291–309. 10.1007/s12687-021-00508-5 33523369 PMC7849219

[B9] Chunara R. Zhao Y. Chen J. Lawrence K. Testa P. A. Nov O. (2021). Telemedicine and healthcare disparities: A cohort study in a large healthcare system in New York city during COVID-19. J. Am. Med. Inf. Assoc. 28 (1), 33–41. 10.1093/jamia/ocaa217 PMC7499631 32866264

[B10] Damschroder L. J. Aron D. C. Keith R. E. Kirsh S. R. Alexander J. A. Lowery J. C. (2009). Fostering implementation of health services research findings into practice: A consolidated framework for advancing implementation science. Implement. Sci. 4, 50. 10.1186/1748-5908-4-50 19664226 PMC2736161

[B11] Danylchuk N. R. Cook L. Shane-Carson K. P. Cacioppo C. N. Hardy M. W. Nusbaum R. (2021). Telehealth for genetic counseling: A systematic evidence review. J. Genet. Couns. 30 (5), 1361–1378. 10.1002/jgc4.1481 34355839

[B12] Dearing J. W. Cox J. G. (2018). Diffusion of innovations theory, principles, and practice. Health Aff. 37 (2), 183–190. 10.1377/hlthaff.2017.1104 29401011

[B13] Dorfman E. H. Brown Trinidad S. Morales C. T. Howlett K. Burke W. Woodahl E. L. (2015). Pharmacogenomics in diverse practice settings: Implementation beyond major metropolitan areas. Pharmacogenomics 16 (3), 227–237. 10.2217/pgs.14.174 25712186 PMC4360986

[B14] Douthit N. Kiv S. Dwolatzky T. Biswas S. (2015). Exposing some important barriers to health care access in the rural USA. Public Health 129 (6), 611–620. 10.1016/j.puhe.2015.04.001 26025176

[B15] Dressler L. G. Bell G. C. Abernathy P. M. Ruch K. Denslow S. (2019a). Implementing pharmacogenetic testing in rural primary care practices: A pilot feasibility study. Pharmacogenomics 20 (6), 433–446. 10.2217/pgs-2018-0200 30983513

[B16] Dressler L. G. Bell G. C. Schuetze D. P. Steciuk M. R. Binns O. A. Raab R. E. (2019b). Implementing a personalized medicine cancer program in a community cancer system. Per. Med. 16 (3), 221–232. 10.2217/pme-2018-0112 31109249

[B17] Fatumo S. Chikowore T. Choudhury A. Ayub M. Martin A. R. Kuchenbaecker K. (2022). A roadmap to increase diversity in genomic studies. Nat. Med. 28 (2), 243–250. 10.1038/s41591-021-01672-4 35145307 PMC7614889

[B18] Fohner A. E. Volk K. G. Woodahl E. L. (2019). Democratizing precision medicine through community engagement. Clin. Pharmacol. Ther. 106 (3), 488–490. 10.1002/cpt.1508 31206610 PMC6692197

[B19] Frydman J. L. Li W. Gelfman L. P. Liu B. (2022). Telemedicine uptake among older adults during the COVID-19 pandemic. Ann. Intern. Med. 175 (1), 145–148. 10.7326/m21-2972 34748380 PMC8845076

[B20] Health Resources and Services Administration (2019). 2019 health center data, Montana state report. data.HRSA.gov. Available: https://data.hrsa.gov/tools/data-reporting/program-data/state/MT/table?tableName=Full (Accessed May 10, 2021).

[B21] Health Resources and Services Administration (2022a). Federal Office of rural health policy. HRSA.gov. Available: https://www.hrsa.gov/rural-health/index.html (Accessed March 20, 2022).

[B22] Health Resources and Services Administration (2022b). Health professional shortage areas (HPSA) dashboard. data.HRSA.gov. Available: https://data.hrsa.gov/topics/health-workforce/shortage-areas (Accessed March 20, 2022).

[B23] Hirko K. A. Kerver J. M. Ford S. Szafranski C. Beckett J. Kitchen C. (2020). Telehealth in response to the COVID-19 pandemic: Implications for rural health disparities. J. Am. Med. Inf. Assoc. 27 (11), 1816–1818. 10.1093/jamia/ocaa156 PMC7337797 32589735

[B24] Hockings J. K. Pasternak A. L. Erwin A. L. Mason N. T. Eng C. Hicks J. K. (2020). Pharmacogenomics: An evolving clinical tool for precision medicine. Cleve. Clin. J. Med. 87 (2), 91–99. 10.3949/ccjm.87a.19073 32015062

[B25] Hundertmark M. E. Waring S. C. Stenehjem D. D. Macdonald D. A. Sperl D. J. Yapel A. (2020). Pharmacist's attitudes and knowledge of pharmacogenomics and the factors that may predict future engagement. Pharm. Pract. 18 (3), 2008. 10.18549/PharmPract.2020.3.2008 PMC7470237 32922573

[B26] Indian Health Service (2019). 2019 fact sheet: Disparities. Available: https://www.ihs.gov/newsroom/factsheets/disparities/ (Accessed May 1, 2022).

[B27] Klein M. E. Parvez M. M. Shin J. G. (2017). Clinical implementation of pharmacogenomics for personalized precision medicine: Barriers and solutions. J. Pharm. Sci. 106 (9), 2368–2379. 10.1016/j.xphs.2017.04.051 28619604

[B28] Maiese D. R. Keehn A. Lyon M. Flannery D. Watson M. (2019). Current conditions in medical genetics practice. Genet. Med. 21 (8), 1874–1877. 10.1038/s41436-018-0417-6 30686822 PMC6752678

[B29] Martin A. R. Kanai M. Kamatani Y. Okada Y. Neale B. M. Daly M. J. (2019). Clinical use of current polygenic risk scores may exacerbate health disparities. Nat. Genet. 51 (4), 584–591. 10.1038/s41588-019-0379-x 30926966 PMC6563838

[B30] Montana Department of Commerce (2020). Income and poverty data for the state of Montana Montana department of Commerce. Available: https://ceic.mt.gov/People-and-Housing/Income-and-Poverty (Accessed May 10, 2021).

[B31] Montana Department of Health and Human Services (2019). Montana shortage area designations. Available: https://dphhs.mt.gov/ecfsd/primarycare/-shortage-area-designations (Accessed May 10, 2021).

[B32] Montana Office of Public Instruction, Division of Indian Education (2020). Montana Indians: Their history and location. Available: https://opi.mt.gov/Portals/182/Page%20Files/Indian%20Education/Indian%20Education%20101/Montana%20Indians%20Their%20History%20and%20Location.pdf (Accessed May 24, 2022).

[B33] Montana State Legislature (2021). House Bill 43: An act generally revising laws relating to telehealth. Available: https://leg.mt.gov/bills/2021/billpdf/HB0043.pdf (Accessed March 30, 2022).

[B34] Morales C. T. Muzquiz L. I. Howlett K. Azure B. Bodnar B. Finley V. (2016). Partnership with the confederated salish and Kootenai tribes: Establishing an advisory committee for pharmacogenetic research. Prog. Community Health Partnersh. 10 (2), 173–183. 10.1353/cpr.2016.0035 27346763 PMC5015644

[B35] Olander M. Waring S. Stenehjem D. D. Taran A. Ranelli P. Brown J. T. (2018). Primary care clinicians attitudes and knowledge of pharmacogenetics in a large, multi-state, healthcare system. Innov. Pharm. 9 (2), 1–12. 10.24926/iip.v9i2.970 PMC6438554 34007697

[B36] Partnership Health Center (2022). Available: https://www.partnershiphealthcenter.com (Accessed January 15, 2022).

[B37] Pfohl M. (2020). FCC grants broadband licenses to 7 of 8 Montana tribes. Available: https://nbcmontana.com/news/local/fcc-grants-broadband-licenses-to-7-of-8-montana-tribes (Accessed June 10, 2021).

[B38] Popejoy A. B. Fullerton S. M. (2016). Genomics is failing on diversity. Nature 538 (7624), 161–164. 10.1038/538161a 27734877 PMC5089703

[B39] Rahawi S. Naik H. Blake K. V. Owusu Obeng A. Wasserman R. M. Seki Y. (2020). Knowledge and attitudes on pharmacogenetics among pediatricians. J. Hum. Genet. 65 (5), 437–444. 10.1038/s10038-020-0723-0 31983733 PMC7109006

[B40] Sandelowski M. (2000). Whatever happened to qualitative description? Res. Nurs. Health 23 (4), 334–340. 10.1002/1098-240x(200008)23:4<334::aid-nur9>3.0.co;2-g 10940958

[B41] Schaaf C. P. (2021). Genetic counseling and the role of genetic counselors in the United States. Med. Genet. 33 (1), 29–34. 10.1515/medgen-2021-2054 PMC11006247 38836204

[B42] Shodair Children’s Hospital (2022). Shodair Children’s hospital. Available: https://shodair.org/(Accessed January 10, 2022).

[B43] Starks H. Trinidad S. B. (2007). Choose your method: A comparison of phenomenology, discourse analysis, and grounded theory. Qual. Health Res. 17 (10), 1372–1380. 10.1177/1049732307307031 18000076

[B44] Stegelmeier J. Nartker C. Barnes C. Rayo H. Hoover R. Boyle J. (2020). Rural community perceptions and interests in pharmacogenomics. Healthc. (Basel) 8 (2), E159. 10.3390/healthcare8020159 PMC7348789 32516951

[B45] Tribal Health Department (2022). Tribal Health Department of the Confederated Salish and Kootenai Tribes. Available: https://cskthealth.org (Accessed January 12, 2022).

[B46] United States Census Bureau (2020). QuickFacts; Montana, United States. United states Census Bureau. Available: https://www.census.gov/quickfacts/fact/table/MT,US/PST045219 (Accessed May 10, 2021).

[B47] Veigle A. (2020). Successful rural digital opportunity fund auction to expand broadband to over 10 million rural. Available: https://docs.fcc.gov/public/attachments/DOC-368588A1.pdf (Accessed June 10, 2021). Americans

[B48] Verbelen M. Weale M. E. Lewis C. M. (2017). Cost-effectiveness of pharmacogenetic-guided treatment: Are we there yet? Pharmacogenomics J. 17 (5), 395–402. 10.1038/tpj.2017.21 28607506 PMC5637230

[B49] Vest B. M. Wray L. O. Brady L. A. Thase M. E. Beehler G. P. Chapman S. R. (2020). Primary care and mental health providers' perceptions of implementation of pharmacogenetics testing for depression prescribing. BMC Psychiatry 20 (1), 518. 10.1186/s12888-020-02919-z 33115428 PMC7594429

[B50] Westby A. Nissly T. Gieseker R. Timmins K. Justesen K. (2021). Achieving equity in telehealth: "Centering at the margins" in access, provision, and reimbursement. J. Am. Board Fam. Med. 34, S29–S32. 10.3122/jabfm.2021.S1.200280 33622814

[B51] Woodahl E. L. Lesko L. J. Hopkins S. Robinson R. F. Thummel K. E. Burke W. (2014). Pharmacogenetic research in partnership with American Indian and Alaska Native communities. Pharmacogenomics 15 (9), 1235–1241. 10.2217/pgs.14.91 25141898 PMC4201360

